# Global Progress in Road Injury Mortality since 2010

**DOI:** 10.1371/journal.pone.0164560

**Published:** 2016-10-11

**Authors:** Peishan Ning, David C. Schwebel, Helai Huang, Li Li, Jun Li, Guoqing Hu

**Affiliations:** 1 Department of Epidemiology and Health Statistics, Xiangya School of Public Health, Central South University, Changsha, Hunan, China; 2 Department of Psychology, University of Alabama at Birmingham, Birmingham, Alabama, United States of America; 3 Urban Transport Research Center, School of Traffic and Transportation Engineering, Central South University, Changsha, Hunan, China; 4 National Key Laboratory of Cognitive Neuroscience and Learning, Beijing Normal University, Beijing, China; Beihang University, CHINA

## Abstract

We aimed to examine progress in global road injury mortality since the initiation of Global Plan for the Decade of Action for Road Safety 2011–2020. We examined annual percent changes in age-adjusted road traffic mortality using data from the Global Burden of Disease Study 2013. Association between changes in road traffic mortality and legislative efforts in individual nations was explored using data from Global Status Reports on Road Safety 2013 and 2015. We found that global age-adjusted mortality, both overall and for user-specific road traffic injuries, decreased significantly between 2010 and 2013 (annual percent change in rates range from -1.43% to -0.99%). Developed countries witnessed a larger decrease than developing countries in both overall and user-specific road mortality (about 2.0–4.6 times). However, there were substantial disparities within developed countries and within developing countries, with some countries seeing large reductions in mortality rates and others seeing none. The annual percent change in road traffic mortality during 2010–2013 was significantly correlated with total national law enforcement score (Spearman *r*_*s*_ = -0.38). We concluded that results highlight the need for continued effort to reduce the burden of road injury mortality, especially in LMIC countries.

## Introduction

Road injury is a significant global public health problem. According to Global Burden of Disease (GBD) estimates, global deaths from road traffic crashes increased 32% between 1990 and 2013, from 1.06 million in 1990 to 1.40 million in 2013 [1]. Road injuries are now the 5^th^ leading cause of global disability-adjusted life years (DALYs) lost, causing approximately 73.3 million life years to be lost [1]. Over 90% of road injury deaths worldwide occur in low- and middle-income countries (LMICs) [2]. Motor vehicle crashes are estimated to cause a total economic loss of over $500 billion, constituting 1–3% of the respective gross national products (GNPs) of the world’s countries [3].

In response to this growing public health challenge, a Global Plan for the Decade of Action for Road Safety 2011–2020 was developed by the United Nations Road Safety Collaboration (UNRSC) in 2010 to integrate and coordinate global efforts to promote road safety [3]. The plan adopts a multi-faceted approach for prevention activities to be conducted at local, national, regional and global levels and the UNRSC has taken measures to promote the implementation of the global action plan in each member country of the United Nations [3]. Experts anticipate 5 million lives could be saved and 50 million injuries could be avoided across the ten-year period if all countries strictly implement the global action plan [3].

The most authoritative monitoring of progress toward the Global Plan of Action goals is conducted by the World Health Organization (WHO) through biannual publications that broadly discuss progress globally [4, 5]. Publications to date indicate progress, with the most recent report suggesting global traffic safety injuries are plateauing and dozens of governments are implementing legislation to reduce traffic injury deaths and injuries. The present report extends the WHO publications by addressing three previously unexamined questions: (a) is global road injury mortality decreasing since the implementation of global action plan, and if so by how much? (b) are developed countries and developing countries achieving equivalent progress in road traffic death mortality? and (c) is national law enforcement assoiated with the extent of recent road safety progress in individual countries? We addressed these questions using data from GBD 2013 estimates and the Global Status Reports on Road Safety 2013 [4] and 2015 [5].

## Materials and Methods

### Ethical issues

This study uses anonymous open-access data and does not involve personal information from individuals. The research was approved by the Medical Ethics Committee of Central South University in Changsha, China.

### Data sources

Road traffic mortality data were collected from the “GBD Compare | Viz Hub”, an online visualization tool provided by the Institute for Health Metrics and Evaluation, University of Washington. The GBD Collaborators use a rigorous process to identify all available data, including the following sources: vital registration documents, verbal autopsy data, mortality surveillance data, censuses, population surveys, hospital records, police records, and mortuary data. The group assesses data quality carefully, including evaluation of completeness, diagnostic accuracy, missing data rates, stochastic variations, and accuracy of probable causes of death [6], and then applies sophisticated modelling strategies to capture spatial and temporal patterns in the data and reduce estimation error. Where possible, objective tests of model performance through out-of-sample predictive validity are conducted to test how well prediction models perform even in settings where no data are available for a particular country [6, 7]. The complex details on data sources, mathematical models, and the methodological limitations of GBD 2013 approaches are described in the appendix of a GBD publication [8].

The GBD 2013 update provides unadjusted and age-adjusted estimates of deaths, mortality, years of life lost, years lived with disability, and DALYs by country, age, sex and cause. The GBD 2013 update includes results for 323 diseases and injuries, 67 risk factors, and 1,500 sequelae for 188 countries [9]. Road injuries are classified into five categories: pedestrian road injuries; cyclist road injuries; motorcyclist road injuries; motor vehicle road injuries; and other road injuries (see detailed International Classification of Diseases codes in GBD-related publications [6, 8]). In this paper, we omit analysis of the “other road injuries” category given its broad scope and low age-adjusted mortality rate in many countries. The GBD 2013 update includes 95% uncertainty intervals of all indicators, a range of values deemed likely to include the correct estimate of health indicators.

Data related to road traffic law enforcement were obtained from the World Health Organization (WHO) Global Status Reports on Road Safety 2013 [4] and 2015 [5], which report national road traffic law enforcement assessment data from the years 2011 and 2014. Both reports examined the presence and enforcement of national legislation related to road safety for speed limits; drink-driving; and the usage of motorcycle helmets, seat-belts and child restraints in 195 countries [4, 5]. There were 13 and 15 countries not participating in the legislation and enforcement surveys in the two reports, respectively, so analyses were omitted for those countries [4, 5]. Enforcement of each road safety law was quantified in the WHO reports using a 0–10 scale yielding total enforcement score ranging from 0 to 50. Low score indicates weak legislative efforts and high score strong legislative efforts. By matching road traffic mortality data from GBD 2013 estimates and data from national legislation and enforcement scores for the five kinds of laws, we obtained relevant data for 145 countries in both 2011 and 2014.

### Data analysis

We first examined annual percent changes in age-adjusted road injury mortality per 100,000 population between 2010 and 2013 in 188 countries. Subgroup analyses were conducted by type of road user and by country development status. Box plots demonstrated country variations in changes in road injury mortality per 100,000 population by road user (pedestrian, cyclist, motorcyclist, and occupant) and development status (developed countries and developing countries, using GBD classifications of development status [1]). Because the crash data were over dispersed, we used negative binomial regression to examine the significance and magnitude of annual percent change in age-adjusted road traffic mortality between 2010 and 2013 [10]. Zero-inflated models were not considered because the methodological precondition was not met. We did not used other advanced models such as random effect count models, and ordered Probit models because the research purpose of this study “examine changes in road safety progress between 2010 and 2013 for each given country” can be achieved using negative binomial regression. Other advanced models may have advantages in reasonably interpreting road safety progresses across countries or accurately forecasting future road traffic mortality when many important factors are available. However, the data of many important factors were unavailable for this study. Annual percent change in mortality was calculated as “regression coefficient*100%”. A significant positive annual percent change indicated a rise of road traffic mortality between 2010 and 2013, while a significant negative annual percent change denoted a fall of road traffic mortality during that period.

Due to lack of open access data for many relevant factors at the national level, we did not run advanced parametric models to quantify the contribution of national legislation effort to road safety progress. Instead, we performed Spearman rank correlation to roughly estimate the association of road safety progress with national legislation effort. Spearman rank correlation is a nonparametric method that describes the direction of the relationship between two variables when the assumption of normal distribution is violated or ordinal variables are involved. In addition, we computed the Wilcoxon rank-sum test to test differences in average national law enforcement scores between developed countries and developing countries for the same year, and between 2011 and 2014. All data analyses were performed using Stata 12.1. “*P*<0.05” was regarded as statistically significant.

## Results

Between 2010 and 2013, worldwide age-adjusted mortality from road traffic crashes decreased 1.12% annually (95% uncertain interval: -1.20%, -1.04%), changing from 20.6 per 100,000 population to 19.9 per 100,000 population (Table 1). Global road traffic mortality for pedestrians, pedal cyclists, motorcyclists, and occupants (drivers and passengers) all decreased significantly over the study time period, with annual percent changes of -0.99%, -1.24%, -1.43%, and -1.09%, respectively. Developed countries witnessed substantially larger decreases both in overall and user-specific road mortality compared to developing countries (all road injuries: -3.23% vs. -0.93%; pedestrians: -3.77% vs. -0.82%; pedal cyclists: -2.43% vs. -1.07%; motorcyclists: -2.96% vs. -1.47%; occupants: -3.08% vs. -0.70%).

**Table 1:** Age-adjusted road injury mortality per 100,000 population between 2010 and 2013 by development status and type of road user.

Status	Category	2010		2013		Annual percent change (%)	
		Rate	95% UI	Rate	95% UI	Percent	95% CI
Global	All road injuries	20.6	(18.8, 21.8)	19.9	(18.4, 21.3)	-1.12*	(-1.20, -1.04)
	Pedestrian	8.2	(6.9, 9.5)	8.0	(6.7, 9.2)	-0.99*	(-1.11, -0.87)
	Pedal cyclist	1.4	(1.1, 1.6)	1.3	(1.1, 1.6)	-1.24*	(-1.54, -0.94)
	Motorcyclist	3.6	(2.9, 4.2)	3.4	(2.8, 4.0)	-1.43*	(-1.62, -1.24)
	Occupant	7.2	(6.3, 8.0)	6.9	(6.1, 7.8)	-1.09*	(-1.22, -0.96)
Developed countries	All road injuries	11.2	(10.7, 12.2)	10.1	(9.5, 11.0)	-3.23*	(-3.48, -2.98)
	Pedestrian	2.9	(2.2, 3.8)	2.6	(1.9, 3.4)	-3.77*	(-4.26, -3.28)
	Pedal cyclist	0.7	(0.5, 0.8)	0.7	(0.4, 0.8)	-2.43*	(-3.41, -1.46)
	Motorcyclist	1.4	(0.9, 1.7)	1.3	(0.8, 1.6)	-2.96*	(-3.66, -2.27)
	Occupant	6.0	(5.2, 7.2)	5.5	(4.7, 6.5)	-3.08*	(-3.42, -2.74)
Developing countries	All road injuries	24.0	(21.4, 25.5)	23.4	(21.2, 25.2)	-0.93*	(-1.01, -0.85)
	Pedestrian	10.2	(8.5, 11.8)	10.0	(8.3, 11.6)	-0.82*	(-0.95, -0.70)
	Pedal cyclist	1.6	(1.3, 1.9)	1.5	(1.3, 1.9)	-1.07*	(-1.37, -0.76)
	Motorcyclist	4.2	(3.3, 5.0)	4.0	(3.2, 4.8)	-1.47*	(-1.67, -1.28)
	Occupant	7.7	(6.5, 8.7)	7.5	(6.4, 8.6)	-0.70*	(-0.84, -0.56)

Notes:

Annual percent change in age-adjusted mortality was estimate based on negative binomial regression.

95% UI: 95% uncertain interval based on GBD 2013 estimates.

95% CI: 95% confidence interval based on negative binomial regression.

*: *P*<0.05.

**Fig 1:**
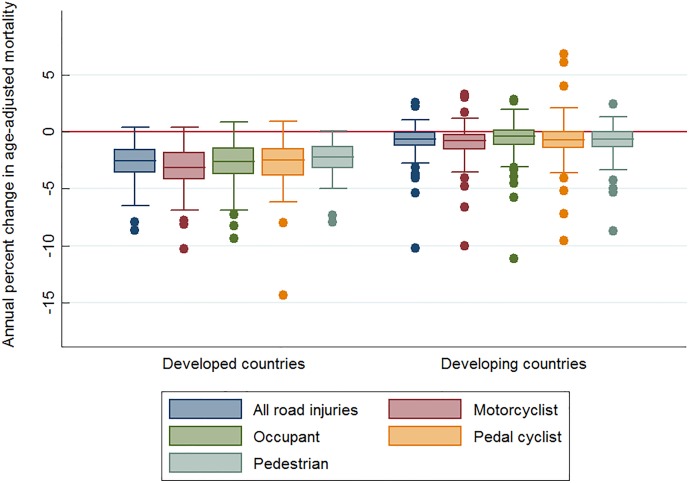
Annual percent change in age-adjusted mortality per 100,000 population in 2010–2013 by type of road user and country. Notes: 1. Annual percent change in age-adjusted mortality was estimated based on negative binomial regression. 2. Mortality data from Global Burden of Disease 2013. (http://www.healthdata.org/gbd/data-visualizations).

Box plots of road injuries by country development status in Fig 1 show: (1) on average, developed countries made more significant progress in both overall and user-specific road injury mortality from 2010 to 2013 than developing countries; (2) within the categories of both developed and developing countries, large variation in progress existed between countries for both overall and user-specific road traffic mortality; (3) a few countries (upper outliers of box plots) experienced substantial increases in overall mortality or in user-specific mortality (all are developing countries); and (4) some countries (lower outliers of box plots) witnessed annual decreases of over 5% in overall mortality or in user-specific mortality. Country-specific data are available in the S1 Table.

**Fig 2:**
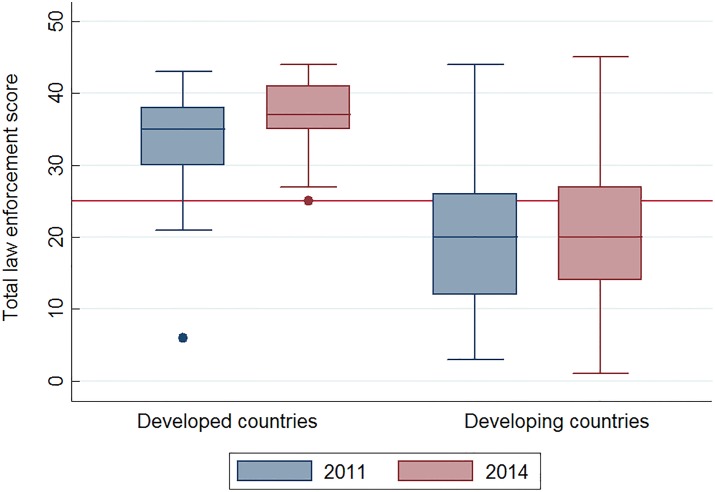
Total enforcement score of five kinds of national road traffic laws in 2011 and 2014 by development status. Notes: 1. National law enforcement scores in 2011 and in 2014 were from the Global Status Report on Road Safety 2013^4^ and 2015^5^, respectively. Total enforcement scores of five kinds of national road traffic laws were calculated, including speed limit law, drink-driving law, motorcycle helmet law, seat-belt law, and child restraint law. Total enforcement score ranged from 0 to 50; 0 points indicated the weakest legislation effort and 50 points mean the strongest legislation effort. 2. Differences in total law enforcement scores was statistically significant between developed countries and developing countries in 2011 (*Z* = 7.21, *P*<0.05) and in 2014 (*Z* = 8.09, *P*<0.05). 3. Change in total law enforcement score between 2011 and 2014 was not significant for developing countries (*Z* = -0.90, *P*>0.05) but was significant for developed countries (*Z* = -2.10, *P*<0.05).

On average, developed countries had higher law enforcement scores than developing countries in both 2011 (median points: 35 vs. 20) and 2014 (median points: 37 vs. 20) (Fig 2). Average changes in law enforcement scores between 2011 and 2014 were not statistically significant for developing countries (*Z* = -0.90, *P*>0.05) but were significant for developed countries (*Z* = -2.10, *P*<0.05). Annual percent change in overall road traffic mortality during 2010–2013 was significantly correlated with law enforcement scores in 2011 (Spearman *r*_*s*_ = -0.38).

## Discussion

Our results suggest global age-adjusted road traffic mortality dropped slightly between 2010 and 2013. On average, developed countries experienced a larger reduction in both overall and user-specific road traffic injury mortality compared to developing countries. Large variations across countries were observed, with some demonstrating substantial reduction in road traffic injuries from 2010–2013, many seeing less reduction, and a few nations even experiencing increases in traffic injury rates. Median scores of law enforcement for five kinds of national road traffic laws were higher in developed countries than in developing countries, and higher law enforcement scores were associated with fewer road traffic injuries.

Our findings offer encouraging evidence concerning the progress of global road safety: slight reductions in overall and user-specific road traffic mortality occurred worldwide between 2010 and 2013. Our research design does not permit us to ascribe the minor reductions causally to the implementation of global action plan [2], but the observed reductions likely reflect the effect of coordinated and similar efforts in many nations, including improved legislation and law enforcement. It is notable also that these decreases occurred in the context of increased motorization in many nations.

Despite generally encouraging results, we also discovered continuing disparities in road traffic mortality across countries. Death rates in some developing countries remain alarmingly high. The newly-released Global Goals for Sustainable Development state a goal “to halve the number of global deaths and injuries from road traffic accidents by 2020” [11]. Society is unlikely to achieve such a goal if road safety efforts lag in certain countries. To date, many countries have failed to fully implement global road safety action plan goals because of economic, political, and other factors [4, 5]. To efficiently promote global road safety, concerted efforts should be made to accelerate road safety progress in those countries.

### Limitations

Our findings are limited in the same way GBD 2013 estimates are limited. Availability and quality of mortality data is poor in many countries, and although GBD 2013 uses highly sophisticated theory-driven modeling to estimate mortality rates, the estimates remain imperfect given the quality of the data available. In addition, the Spearman correlation analysis is imperfect because it fails to control for any number of moderating factors that may influence the association. Future research should work to control covariates in multivariate models that are strictly controlled with data available in individual countries or regions to quantify the contribution of national legislation efforts to road safety progress.

## Supporting Information

S1 Table Annual percent change in age-adjusted road traffic mortality per 100,000 population and 95% uncertain interval (all road injuries, 2010–2013). (XLSX)
